# Transplantable Melanomas in Hamsters and Gerbils as Models for Human Melanoma. Sensitization in Melanoma Radiotherapy—From Animal Models to Clinical Trials

**DOI:** 10.3390/ijms19041048

**Published:** 2018-04-01

**Authors:** Martyna Śniegocka, Ewa Podgórska, Przemysław M. Płonka, Martyna Elas, Bożena Romanowska-Dixon, Małgorzata Szczygieł, Michał A. Żmijewski, Mirosława Cichorek, Anna Markiewicz, Anna A. Brożyna, Andrzej T. Słominski, Krystyna Urbańska

**Affiliations:** 1 Department of Biophysics, Faculty of Biochemistry, Biophysics and Biotechnology, Jagiellonian University in Kraków, 31-007 Kraków, Poland; przemyslaw.plonka@uj.edu.pl (P.M.P.); martyna.elas@uj.edu.pl (M.E.); gosia.szczygiel@uj.edu.pl (M.S.); 2 Department of Ophthalmology and Ocular Oncology, Medical College of Jagiellonian University in Kraków, 31-007 Kraków, Poland; bozena.romanowska-dixon@uj.edu.pl (B.R.-D.); annamarkiewicz@interia.pl (A.M.); 3 Department of Histology, Medical University of Gdansk, 80-210 Gdańsk, Poland; mzmijewski@gumed.edu.pl; 4 Department of Embryology, Medical University of Gdansk, 80-210 Gdańsk, Poland; cichorek@gumed.edu.pl; 5 Department of Tumor Pathology and Pathomorphology, Faculty of Health Sciences, Nicolaus Copernicus University Collegium Medicum in Bydgoszcz, Oncology Centre-Prof. Franciszek Łukaszczyk Memorial Hospital, 85-796 Bydgoszcz, Poland; anna.brozyna@cm.umk.pl; 6 Department of Dermatology, Comprehensive Cancer Center Cancer Chemoprevention Program, University of Alabama at Birmingham, Birmingham, AL 35294, USA; 7 VA Medical Center, Birmingham, AL 35294, USA

**Keywords:** melanoma, melanins, Bomirski hamster melanoma, ocular melanoma, human melanoma, X-rays, neutrons, proton beam irradiation, radio-chelation therapy, radio-photo-therapy, chemo-radiotherapy, tumor vessels

## Abstract

The focus of the present review is to investigate the role of melanin in the radioprotection of melanoma and attempts to sensitize tumors to radiation by inhibiting melanogenesis. Early studies showed radical scavenging, oxygen consumption and adsorption as mechanisms of melanin radioprotection. Experimental models of melanoma in hamsters and in gerbils are described as well as their use in biochemical and radiobiological studies, including a spontaneously metastasizing ocular model. Some results from in vitro studies on the inhibition of melanogenesis are presented as well as radio-chelation therapy in experimental and clinical settings. In contrast to cutaneous melanoma, uveal melanoma is very successfully treated with radiation, both using photon and proton beams. We point out that the presence or lack of melanin pigmentation should be considered, when choosing therapeutic options, and that both the experimental and clinical data suggest that melanin could be a target for radiosensitizing melanoma cells to increase efficacy of radiotherapy against melanoma.

## 1. Introduction

Malignant melanoma is one of the most notorious and deadly human tumors. It has been known since at least the fifth century B.C., when it was mentioned by Hippocrates of Cos [1]. Its most characteristic biological feature is the ability to produce and store melanin [1], which in turn is also responsible for important features of the tumor, making it resistant to many modes of therapy [2].

There are numerous papers exploring the models of murine melanomas. The present review intends to summarize the work on less popular animal models in melanoma research, i.e., Syrian golden hamsters (*Mesocricetus auratus* Waterhouse 1839) and Mongolian gerbils (*Meriones unguiculatus* Milne-Edwards 1867). These laboratory animals and their melanomas reveal quite unique features which in some aspects make them akin to human melanomas. The research has been carried out for over 50 years in several research centers, mainly in Poland and USA, largely using models developed locally in Gdansk and Krakow (Bomirski hamster melanoma BHM [3,4,5,6]), and gerbil melanoma Zeman UJ90 [7,8], which makes this compilation of research quite unique. A particular emphasis has been placed on radiotherapy and melanin as factors determining the effectiveness of melanoma therapy.

## 2. Melanin Functions and Their Radioprotective Role in Melanoma

Melanoma originates from melanocytes, which are responsible for generating melanin [9]. This polymer is produced in melanosomes, organelles coming from lysosomal lineage, and transferred to other cells in the mammalian skin, mainly to keratinocytes [10]. This transfer can be disturbed in melanomas, which may either store large amounts of melanin in the cytoplasm or secrete it to the environment [8,11,12]. Consequently, it leads to intra- or extracellular accumulation of melanin or its precursors, which can be toxic [13,14]. During the process of melanogenesis, such intermediates are generated, which may accelerate the induction of secondary mutations in melanoma cells [15]. This can be one of the reasons for which a melanotic phenotype in late-stage melanoma is connected with poor prognosis [16]. Moreover, the signaling pathways responsible for melanin synthesis are partially common with those involved in neoplastic transformation [17,18,19,20]. As a result, in a melanogenically active cell, every mutation in genes coding regulatory factors, such as the cAMP response element-binding protein (CREB) [21], PI3K/p70 [22], c-KIT [23], (neuroblastoma RAS viral oncogene homolog) (NRAS) [24,25], or (serine/threonine-protein kinase B-raf) (BRAF), becomes automatically expressed [26,27,28], manifesting itself in the pigment phenotype, and in gaining neoplastic features at the same time [29].

On the other hand, the generation of melanin itself is protective for the body and the cell. The production of melanin is of a clearly adaptive value. Thus, it provides animal camouflage, and it protects the body against different type of radiation including UV protection spectrum [30,31]. Being a macromolecule of amorphous structure, it often reveals properties of inorganic rather than organic substances [32]. Its spectrum of absorption resembles inorganic material [33], as devoid of characteristic maxima of absorption, and monotonously decreases from UV towards longer waves, and for infra-red irradiation melanin becomes almost transparent [33,34]. This broadening of spectrum is responsible for its brown to black coloration, and also makes it a good photoprotector because of a high efficiency of conversion of the absorbed irradiation into heat [32,34]. Melanin contains numerous carboxylic groups of various degrees of protonation, and quinone/hydroquinone groups of various degrees of oxidation, containing also semiquinones responsible for its paramagnetic properties [32,35,36]. All that makes melanin a powerful buffer able to reversibly protonate, and a redox-buffer able to reversibly oxidate and recombine with external free radicals (e.g., produced by radio- or photolysis of water in the cytoplasm [37,38]). The complicated, irregular inner structure makes melanin an efficient ion-exchange resin able to reversibly adsorb metal cations (zinc, calcium, iron, copper, manganese, etc.), organic compounds, gases, and water [32,36,39,40].

Despite its resistance to acid hydrolysis, melanin undergoes degradation in the body, probably with the engagement of the reduced nicotinamide adenine dinucleotide phosphate (NADPH)-dependent oxidoreductases (NOX), and/or the visible light and UV [41,42]. The substances adsorbed in the past may be released back in the process of melanin degradation, and made active with a delay, some of them (e.g., iron or copper) being photochemically or redox-active themselves. This is responsible for melanin toxicity and phototoxicity, stronger for pheomelanin than for eumelanin [19,38,43,44,45].

**Figure 1:**
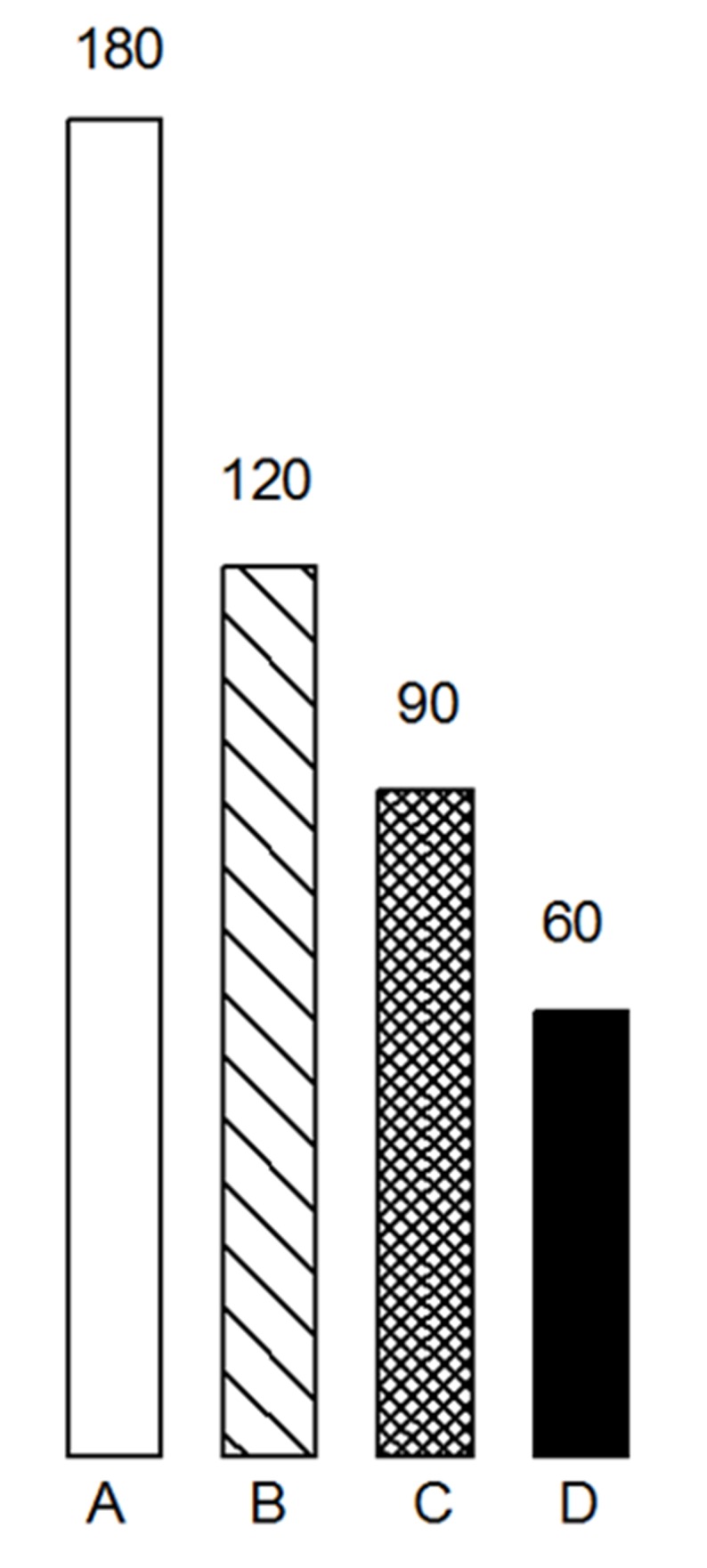
The presence of melanin in polymethyl methacrylate (PMM) inhibits the decay of free radical signal, induced by X-rays. **A**—450 mg of pure PMM, **B**—450 mg of PMM with 2 mg of melanin, **C**—450 mg of PMM with 4 mg of melanin, **D**—450 mg of PMM with 6 mg of melanin. Figure based on [47]. Copyright 1975 IAEA.

The high accumulation of paramagnetic centers in the molecules of melanin was suggested to enable this polymer to interact with radiation-induced free radical species as a radical scavenger. The comparative studies on the sensitivity of animal cells to low-linear energy transfer (LET) radiation support the view that melanin content of the cell can affect its radiation response [46]. Polymethyl methacrylate (PMM) mixed with melanin in different concentrations was irradiated with 75 Gy to study the X-ray-generated free radical signal. It was demonstrated that oxygen-centered polymer signal was decreasing faster in the presence of increasing concentrations of synthetic melanin [47], directly showing the protective role of melanin addition (see Figure 1). Similar reactions can be found if dry collagen is used as a matrix instead of PMM [47].

In addition to the interaction of melanin with radiolytic products, another purely chemical process which may contribute to the overall level of cellular radioresistance was reported [48]. It has the nature of nonenzymatic binding of molecular oxygen by all types of natural and synthetic melanins, and not of physical sorption by these polymers, as can be deduced from the temperature dependence of the process. In melanoma cells, melanin is enclosed in melanosomes, and separated from the rest of the cell. Oxygen is a well-known radiosensitizer. Thus, any process which sequestrates it in the cell, and decreases its concentration in the direct neighborhood of organelles sensitive to radiation damage (mitochondria, the nucleus), should be associated with an enhancement of radioresistance.

There are three main mechanisms of radioprotection of cells by melanin:

1. Melanin is able to absorb the radiation itself (e.g., the UV irradiation [49,50], ionizing radiation [51,52]) due to the broadened spectrum of absorption and efficient internal conversion of the absorbed energy [32,34];
2. Melanin recombines with products of radio- and photolysis of water [37], and other compounds of melanotic cells [32,53];
3. Melanin consumes and adsorbs oxygen, and the process of melanin synthesis consumes significant amount of the co-substrate—oxygen (e.g., for tyrosinase [54], or phenylalanine hydroxylase [55], etc.).

As the synthesis of new melanosomes is continuous in pigmented cells [8,19] this makes the stationary intracellular oxygen levels low, thus rendering the whole cell less vulnerable to irradiation (as oxygen is an efficient radio- and photosensitizer [56]).

Despite the many mechanisms of protection, the potential toxicity of melanin may in some cases outweigh the benefits of its presence in the cell as a radioprotector [57]. For example, melanin toxicity is synergic with manganese (II) ions and facilitates DNA damage in neurons [58]. Recently, it has been shown that the high-symmetry manganous complexes are responsible for radioresistance in many organisms, while complexes of low symmetry may radiosensitize the cells [59]. As the latter are created in active centers of enzymes (such as superoxide dismutases), radiation resistance may not depend on the genetically encoded ability to minimize oxidative stress, but on the actual nutritional status of the cell [59]. This may, to some extent, explain the synergistic effect of melanin and manganese (II) in cell radiosensitization, because melanin has been convincingly proven to sequestrate Mn^2+^ cations by means of creating low-symmetry complexes [40].

## 3. Hamster as a Model of Spontaneously Occurring Melanoma

The full history of the discovery, description, and domestication of the Syrian golden hamster (*Mesocricetus auratus* Waterhouse 1839) for laboratory purposes, and as pets, is exciting and worth a separate paper (supplemented with the newest discoveries, [60]). Caught near Aleppo, Syria, in 1930 [60], the golden hamsters turned out to be perfect laboratory rodents for two main reasons: easy and instant taming of the wild animals, and their extreme resistance to inbred despite a high genetic drift and founder effect [60]. As a result, the laboratory population reveals low polymorphism in the histocompatibility loci [9,61,62]. Consequently, the animals are able to maintain allogenic transplants, and in particular the ones of tumor tissues, in a higher percentage of cases than the wild population. Another consequence is a particular susceptibility of laboratory hamsters to human viral infections [61]. The spectrum of application of those animals as laboratory models of human diseases is thus unexpectedly wide. Helen Valentine et al. [63] described at least 17 types of human pathologies, including chronic obstructive pulmonary disease (COPD), atherosclerosis, diabetes mellitus, or amyloidosis, which have been studied using the Syrian hamster model. Hamsters are also used to study the Ebola virus [64]. It is worth reminding that the primary reason for accepting the golden hamster as a laboratory animal was its particularly high sensitivity to infections with *Salmonella typhimurium*, which raised researchers hope of developing a vaccine [65]. Of note, even though the works are on hold now, as the microbe is now explored as part of an alternative strategy in cancer treatment [66]. Another important feature of the Syrian hamsters is the variability in hair-coat coloration phenotypes, with numerous color mutants [67], which makes them particularly interesting objects of genetic studies and studies on the influence of hair color phenotypes on melanoma development [68].

As models for human melanoma, the Syrian hamsters turned out to develop spontaneous and chemically-induced melanomas easily, and with particular metastatic potential, especially in the first group [69]. The animals treated with carcinogens were found to develop both nests of extrafollicular melanocytes around selected pilo-sebaceous units [70] and benign pigmented nevi adjacent to the basement membrane of the epithelium [69]. They also revealed amelanotic melanocytes at the dermo-epidermal junction of the skin [71]. All of these atypical groups of melanocytes, as well as dermal melanocytes might be able to transform into melanomas [69,70,72,73]. Therefore, the actual origin of various types of tumors is not yet fully understood. Interestingly, the hamsters are resistant to UV-induced melanomagenesis [69,74]. Several in vivo and in vitro models of melanoma in golden hamster have been developed. The most important spontaneous melanomas are Greene melanoma models [75] (used e.g. as xenotransplants to the anterior chamber of the rabbit eye to study the effectiveness of hyperthermia [76] and photodynamic therapy [77]), Fortner’s hamster melanoma [71,78] (known to present as ascites in the peritoneal cavity [79,80]), and Bomirski melanoma [3], described in details in the next chapters.

### 3.1. Bomirski Hamster Melanoma

**Figure 2:**
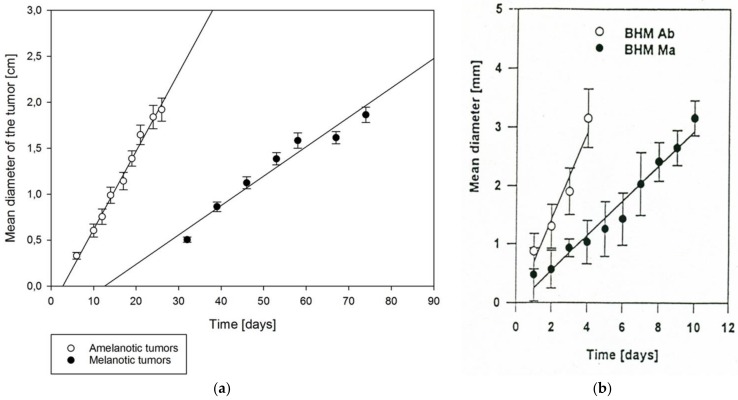
(**a**) The kinetics of Bomirski hamster melanoma (BHM) Ab and Ma tumors growth after subcutaneous implantation in Syrian Hamsters. White dots indicate amelanotic tumors growth in time, whereas black dots indicate melanotic tumors growth in time. Amelanotic tumors begin to grow 3 days after implantation, whereas melanotic tumors start growing 12 days after implantation. (**b**) The growth kinetics of BHM Ab [○] (*n* = 9) and BHM Ma [●] (*n* = 14) in the hamster eye. Each point represents the mean ± SEM [84].

The Bomirski hamster melanoma (BHM) model consists of two basic melanoma lines: melanotic Ma (black) and amelanotic Ab (white), with the latter being an example of tumor progression [5,6,81,82]. The original melanotic Ma line was derived from a spontaneous melanoma of the skin (located near the nose) that had appeared in a male Syrian (golden) hamster in 1959, and which has been maintained by serial transplantation among random-bred animals. The amelanotic melanoma line (Ab) originated in 1963 from the Ma form by spontaneous alteration, which included loss of the ability to produce melanin pigment, marked increase in growth rate, loss of the ability to metastasize and decrease of the survival time [4,5]. Once established, these melanomas possessed a considerable degree of phenotypic stability over decades of passaging [4,5]. The two lines have been successfully transferred allogenically in hamsters for over fifty years. As a result of the faster growth of the Ab line, its transplantation interval is shorter than that of the native Ma line, although with years of passaging it has been decreasing for both lines [5], as further documented by a shorter transplantation and latency period and decrease of minimal tumor cells required for transplantability [6,83,84]. The subcutaneous growth of tumors is compared in Figure 2a.

Metastases of the Ma line to lungs and regional lymph nodes have been observed from the beginning, while the Ab line formed metastases in kidneys and liver only twelve years after the first transplantation [5].

The Ab amelanotic melanoma lost the ability to synthesize melanin as a result of a block in melanosomes biogenesis, but has retained the tyrosinase activity, although at a markedly lower level than that in the Ma line [4,5,6]. The detectable tyrosinase activity is unique among animal amelanotic melanomas making the Ab line similar to human amelanotic forms expressing tyrosinase activity. The loss of melanin synthesis was accompanied by changes in many biological features, including a faster tumor growth rate, shorter animal survival, and changes in the ultrastructure of cells. The loss of ability to produce melanin pigment was reversible, since the cells started to produce melanin de novo when incubated in media high in tyrosine or other melanin precursors [85,86,87,88,89,90], with complex phenotypic changes dependent on the type and concentration of the melanin precursors used [81,82,87,91,92,93,94,95,96,97,98,99,100,101,102].

The histological and ultrastructure analysis showed that Ab melanoma cells, besides the absence of premelanosomes, have an extensive Golgi apparatus, abundant ribosomes, and their plasma membrane structure and content of DNA is changed in comparison to Ma melanoma (see details in Table 1). In addition, Ma and Ab differentially influence the immunological system through modified antigenicity, immunogenicity, and cytokine secretion (details in Table 1).

Apart from the higher proportion of cells in S/G2/M phases (Table 1), the Ab line also has a decreased ability to undergo spontaneous apoptosis in comparison to the Ma line [103,104]. However, the Ab line is very sensitive to camptothecin-induced death [103,104] and showed significantly higher radiosensitivity in comparison to melanotic Ma melanoma [105].

Basic metabolic parameters indicate that the Ab line is different in terms of the type of energy-yielding metabolism, including glycolysis and mitochondrial oxidation, and other metabolic parameters, including the pentose phosphate pathway, from the native melanotic melanoma line [84,92,105,106,107,108]. The native melanotic Ma melanoma cells have higher oxygen consumption than the amelanotic Ab line [106], while amelanotic melanoma has a higher rate of aerobic and anaerobic glycolysis [84], and higher basal mitochondrial transmembrane potential ΔΨ in comparison to Ma line cells [109] (details in Table 1 [6,84,106,107]). These metabolic differences, defined by the presence or absence of melanin pigmentation, were further mechanistically substantiated by analyzing them during induction or stimulation of melanogenesis in cell culture or in isolated cells [84,92,108].

Since the establishment of the BHM lines, each BHM melanoma line has maintained phenotypical stability (melanin production, growth rate, morphology) for over fifty years of transplantation. The comparative biological characteristics of melanotic Ma and amelanotic Ab BHM are presented in Table 1.

### 3.2. MI Melanoma and Ab-455

A third variant of transplantable Bomirski melanoma was established in 1976 from a partially depigmented passage 104 of Ma melanoma, and then transplanted subcutaneously in hamsters [6,126]. It differed from the parental Ma melanoma in its higher tyrosinase activity, lower pigmentation level, ability to produce pheomelanin, and slightly slower growth rate, with other parameters similar to the Ma melanoma [126]. Selection for more pigmented tissue during transplantation of MI melanoma generated another variant that differed from the MI melanoma only in terms of its higher pigmentation level [127]. Additional line Ab-455, transplantable in hamsters, was derived from the in vitro cell line originating from the primary culture of Ab melanoma [88]. That amelanotic tumor was tyrosinase negative, grew significantly slower than the parental Ab melanoma, and had a different metastasis pattern that was similar to the Ma melanoma. Interestingly, during serial transplantation, a rapid acceleration of Ab-455 growth occurred, rendering it similar to the original Ab melanoma [88].

### 3.3. Radiosensitivity of BHM Growing in the Skin

#### 3.3.1. Effects of Low-LET Radiation

A striking difference between the radiosensitivity of the pigmented (BHM Ma) and nonpigmented Bomirski hamster melanoma (BHM Ab) was observed in the early 70’s (unpublished). Subcutaneously growing tumors were irradiated with 48 Gy of fractionated X-rays (two times 7 Gy and two times 5 Gy every 24 h, then repeated after 6 days, 50 kV, 25 mA, Al 1 mm, 4.98 Gy/min). The growth of both BHM Ma and BHM Ab tumors was inhibited, but amelanotic tumors disappeared markedly faster [128].

The higher radioresistance of pigmented cells was verified later in a more elaborate experiment involving irradiating melanoma cells in vitro, and determining the survival fraction in vivo. That step was designed in order to check if the higher radioresistance of pigmented lines originated at the tissue or at the cellular level.

**Figure 3:**
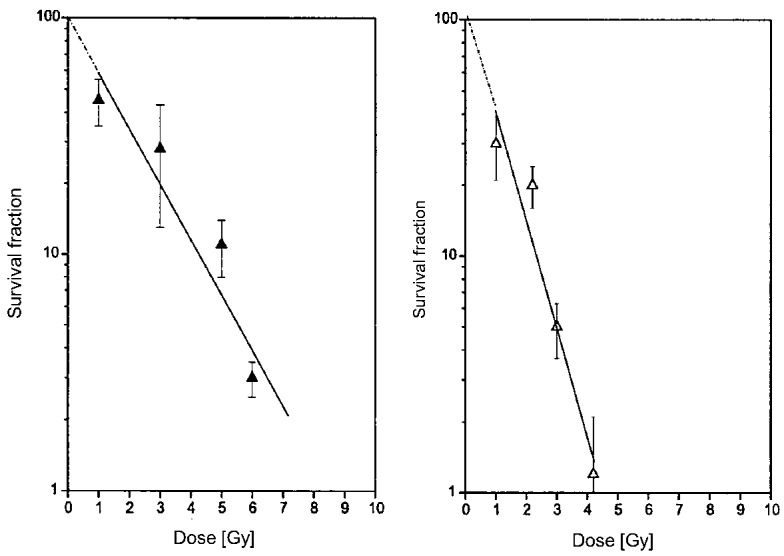
Pigmented cells were more resistant to X-rays than unpigmented Ab cells. The cells were irradiated in vitro, and immediately after implanted subcutaneously into hamsters, always using the same number of cells (10^6^). The average rate of tumor growth was determined for each dose, and the survival fraction of irradiated cells was calculated from a set of tumor growth curves, where the tumor was initiated with various cell numbers [129]. Copyright 1984 Gurbiel, R.

Cells were irradiated in vitro, and immediately after implanted subcutaneously into hamsters, always using the same number, 10^6^ cells. The average rate of tumor growth was determined for each dose. The survival fraction of irradiated cells was calculated from a set of tumor growth curves, where the tumor was initiated with various cell numbers. Figure 3 shows that pigmented cells were 2.4 times more radioresistant than unpigmented BHM Ab. The mean lethal dose was 4.8 Gy for BHM Ma and 2.0 for BHM Ab [129].

#### 3.3.2. Radio-Chelation Therapy

Radio-chelation therapy is based on the combination of radiotherapy with a parallel use of chelating drugs as radiosensitizers. The latter may exert no oncostatic effect by themselves. The chelator Edathamil calcium-disodium (ECD) was combined with 20 Gy of X-rays, delivered as 4 Gy every 5 days. The highest concentration of the chelator in the tumor tissue could be achieved upon topical application of a 10% ointment of ECD over subcutaneously (s.c.) implanted hamster melanoma. Tumor growth was inhibited for 33 days and tumor volume at day 33 was three times smaller than the control, i.e., vehicle plus radiation (Lukiewicz et al., data not published). 

#### 3.3.3. Effects of Neutrons

**Figure 4:**
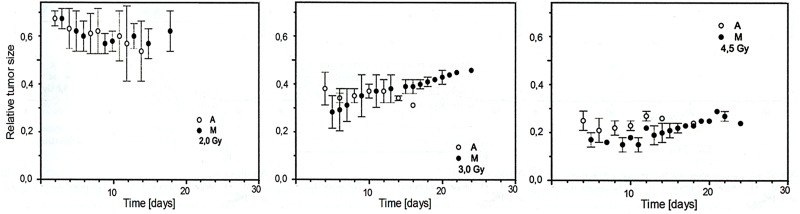
No difference in radiosensitivity between Ab and Ma cells treated with neutrons [130]. Copyright 2000 Urbanska, K.

The next step was to check the radiosensitivity in the same in vitro-in vivo model against high-LET radiation, i.e., neutrons. As there is no oxygen effect in high-LET radiation, both sub-lines were expected to exhibit the same radiosensitivity. Indeed, experiments demonstrated that the then striking difference in radiosensitivity between Ma and Ab lines, clearly visible for low-LET radiation (X-rays), disappeared for the irradiation of the two tested sub-lines with 5.5 MeW neutrons (Figure 4)**.**

### 3.4. Radiosensitivity of BHM Tumors Transplanted in the Eye

Several animal models of ocular melanoma were proposed, including implantation of skin melanoma into the eye, such as the Greene melanoma, B16 [131], or human uveal melanoma in the nude mouse eye [117,132,133,134,135]. Hu successfully established mouse xenografts in the choroid of an immunosuppressed rabbit using B16F10 cell line [134]. Our ocular tumor model of melanoma was obtained by implanting small pieces of BHM, freshly excised from the cutaneous tissue, into the eye of the Syrian hamster (*Mesocricetus auratus*) [135]. Tumor fragments sized 0.4–1.0 mm were implanted into the anterior chamber (AC) of the eye. During the first 2 to 3 days, disappearance of the implants was observed, followed by the appearance of iris tumors after 4–6 days in the case of BHM Ab, and after 8–10 days in the case of BHM Ma. When the AC was completely filled with the tumor mass, the eye was enucleated, and the animals were observed for metastases, developing within 20–30 days in the lung. Melanoma cells growing in the iris in the form of nodules infiltrated all surrounding tissues, and the ciliary body in particular, and always remained pigmented. The distant metastases, assessed macroscopically, were encountered in the lungs (after 48 days in 100% of animals), and sometimes in the kidneys (after 48 days only sporadically) as well as in the regional lymph nodes which were also clearly enlarged [5,136].

**Figure 5:**
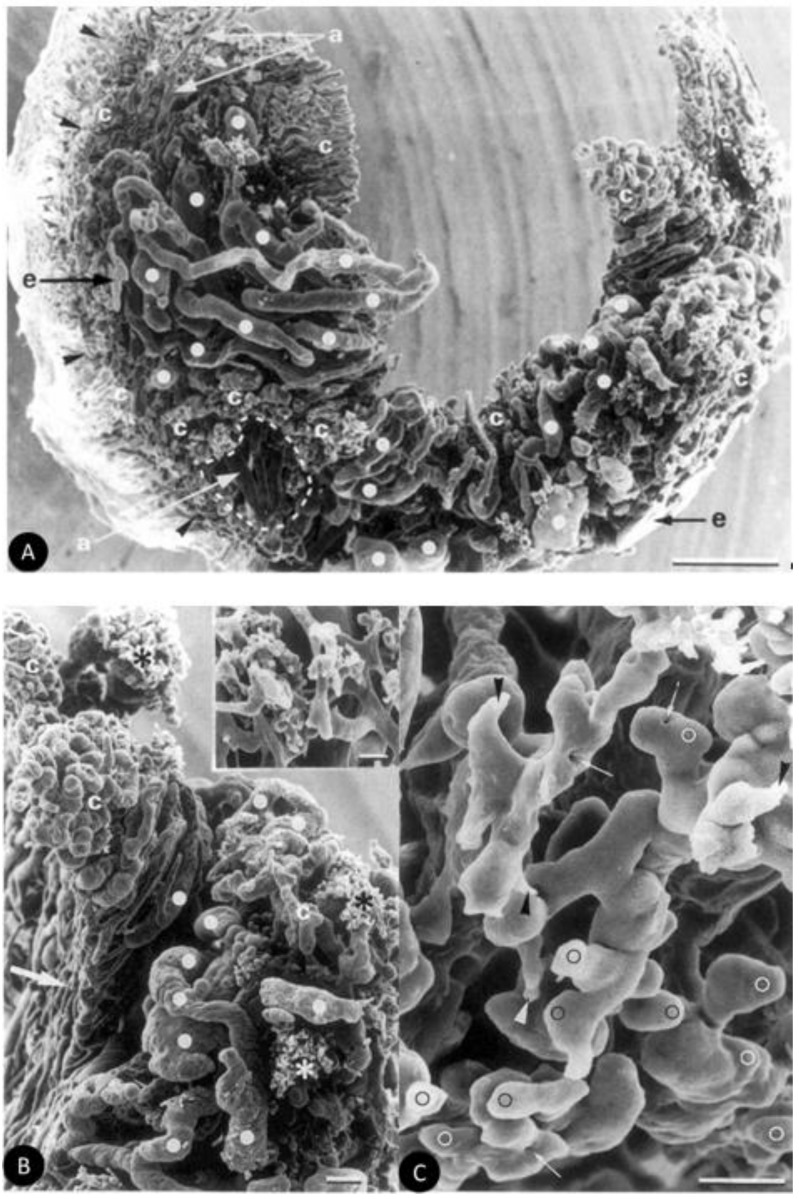
(**A**) Tumor vasculature cast revealing a missing vessel hierarchy and heterogenous vascular density. Strongly dilated venous vessels (full white circles) and capillaries (**c**) predominate over few arterial feeders (**a**) Nodular, nest-like, avascular areas, surrounded by tufts of capillaries with short terminal branches, are marked with a dashed line. The external perimeter of tumor vasculature is indicated with arrowheads. Extravasation of resin is also visible (**e**) Bar = 500 µm. (**B**) Fragment of intratumor vasculature showing strongly dilated venous vessels (full white circles) interconnected with loops formed by wide sinusoidal capillaries (**c**) Tufts of capillaries with short terminal branches are indicated (asterisks) and shown in higher magnification (inset). Note also the posterior vascular layer of the iris (arrow). Bar = 100gm and 50 gm, respectively. (**C**) Vascular sprouts (arrowheads) and globular outgrowths (white circles) on the proliferating, dilated tumor capillaries. Note also the tiny holes (arrows) typical of the intussusceptive angiogenesis. Bar = 500 µm. Reproduced with permission from Annals of Anatomy [137]. Copyright 2001 Elas, M.

The vasculature of BHM tumors growing in the eye was mainly induced from the anterior capillary and antero-venular layers of the iris. As it was revealed by scanning electron microscopy of vascular corrosion casts (Figure 5), the tumor vasculature was characterized by pronounced tortuous courses of the blood vessels with uneven contours and variable diameters. All vessels were highly irregular and heterogeneous, with many embolizations, fenestrations, and sprouting. Venules and sinusoidal capillaries, exhibiting heterogeneous intra-tumor density, were intensively interconnected. Avascular areas were also seen. The presence of numerous nodular outgrowths, varying in size, on the surface of dilated venules and venous vessels represents morphological evidence for the continuous remodeling of tumor vasculature. The observed features of the vascular system seem to provide a pathway for further tumor expansion [137].

Although BHM is a cutaneous melanoma, the development of spontaneous metastases is an advantage of the model [136]. Also, unlike the rabbit model, BHM melanoma is allotransplanted with hamsters being both the donor and the recipient of the graft. This eliminates immunological complications such as graft rejection, which can occur after the transplantation of hamster melanoma (Greene melanoma) into the rabbit eye.

#### 3.4.1. Effects of Low-LET Radiation

Since 2000, the hamster model of melanoma located in the eye has been applied to study distant metastases studies [130]. Two sub-lines differing in their melanin content were compared with regard to their radiosensitivity to ruthenium-106 (^106^Ru) radiation. Tumors growing in the iris were treated with 3, 6, or 10 Gy of ^106^Ru administered as a single dose or in four fractions at 24 h intervals. Dose-dependent delay of tumor growth was observed in both melanomas. Following the treatment with a dose of 6 Gy, the amelanotic (BHM Ab) tumors grew 2.6 times slower, and the melanotic (BHM Ma) tumors 1.4 times slower than the untreated ones. Exposure to β-radiation from ^106^Ru did not significantly affect either the number or the size of metastases, except at a dose of 10 Gy, where a statistically significant decrease in the number of metastases was found in the melanotic sub-line (BHM Ma) [138]. Histological analysis showed signs of tumor blood vessel damage such as endothelial cell swelling, erythrocyte extravasation, and tumor necrosis. These signs increased with the rising dose of β-radiation. Change of fractionation from four equal doses to a boost dose of 4 Gy, followed by 3 × 2 Gy, caused a complete inhibition of metastases for 70 days (unpublished data).

#### 3.4.2. Radiotherapy Using Proton Beam Irradiation

**Figure 6:**
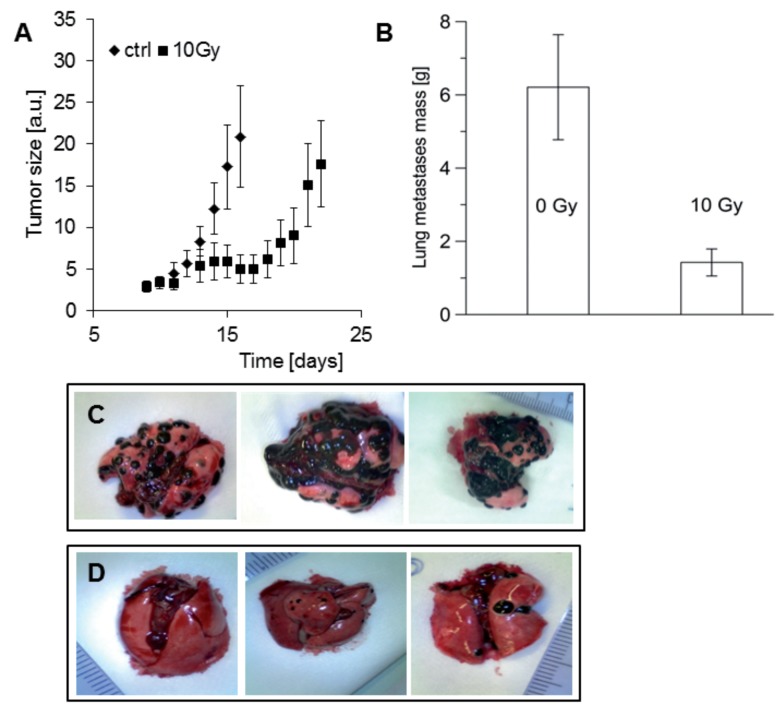
(**A**) Inhibition of BHM melanoma tumor growing in the hamster eye, irradiated with a proton beam at a single dose of 10 Gy (*n* = 7, black square), as compared with the untreated control (*n* = 6, black diamond). (**B**) The mass of lung metastases decreased 4.35 times as a result of the proton beam irradiation (10 Gy) of BHM melanoma tumor growing in the hamster eye (*p* = 0.0052). Average mass with SEM is shown. The number of control animals was six, and the number of irradiated animals was seven. Representative isolated lungs with metastases from untreated (**C**) and irradiated (**D**) animals. Reproduced with permission from [138].

A single dose of 10 Gy of proton beam irradiation delayed the growth of BHM Ma melanoma in the hamster eye by 10 days [138]. Albeit the inhibition of the implanted tumor growth was moderate, proton therapy noticeably reduced the mass of the metastases in the lung in comparison with untreated tumors (Figure 6). On average, 10 Gy of proton irradiation diminished the mass of metastases 4.35 times, even though there was a significant spread between individual animals (Figure 6). These results are in agreement with data presented for osteosarcoma [139]. Likewise, proton beam irradiation decreased cell migration and invasion in a dose-dependent manner and strongly inhibited matrix metalloproteinase 2 (MM-2) activity in highly aggressive HT 1080 human fibrosarcoma cells in vitro [139]. Similarly, it was shown that in vitro models, the adhesion, migration, invasion, and the level of expression or activity of molecules related to metastases, such as αVβ3, β1 integrin, and MMP-2, were all decreased, even after treatment with small doses of proton beam [140].

#### 3.4.3. Radio-Phototherapy

Studies on the effects of photodynamic therapy (PDT) combined with γ-radiation on the BHM transplanted into the eye were especially important as both of the methods are non-invasive and thus prevent surgical intervention. The main advantage of ionizing radiation is the deep penetration of radiation into the tissue, where it inhibits cell division. PDT, on the other hand, targets mainly tumor cells, but visible irradiation, using the preferred excitation wavelength for the MC540 sensitizer, penetrates only partly into the tumor tissue. Combining PDT with a very low-dose rate of γ-irradiation was found to lead to tumor inhibition [141]. Although the histological damage was severe for both lines, the non-pigmented BHM Ab line was more sensitive and responded better than the pigmented melanoma. The most significant inhibition was obtained when both the γ-radiation and PDT were delivered in doses spread over time. Such treatment resulted in 6 weeks of inhibition, a far greater length of time than the inhibition period observed after a single treatment (2 days for non-pigmented cells and 4 days for the pigmented ones).

The significant increase in effectiveness with the four divided combined treatment doses may be due to radiation-induced depletion of the viable stem cells. That finding agrees with the results obtained in the rabbit eye for Greene melanoma [94]. The cumulative results indicate that MC540-mediated PDT in combination with ionizing radiation has significant effects on the rapidly growing melanoma in the eye [142].

## 4. Gerbils as Animal Models for Chemically-Induced Melanomas

The Mongolian gerbil belongs to the family Muridae, subfamily Gerbillinae, order Rodentia [143]. Gerbils are small rodents that occur naturally in the desert regions of Northeast China, Eastern Mongolia and the steppes of Russia [144], living in small colonies in extended burrow systems [145]. They have several phases of twenty-four-hour activity; the two most active periods are just after dawn, and around dusk, but many gerbils remain active throughout the day [146].

Gerbils have been used for scientific purposes since the 1880s, beginning with the research on tuberculosis; they also played a significant role in bilharzia research during the 1950s–1960s [147]. Due to its characteristic behavioral and physiological features, gerbils were used in a wide spectrum of research, covering a variety of research fields, including behavioral investigations [148], biological and behavioral processes of aging [149], epilepsy [150], infectious diseases [151,152,153], dermatitis, neurology research, audiometry and sound effect, coat color genes [154], melanin and tyrosinase activity [155], and others. The gerbil is an important laboratory animal in oncology research [8,156,157,158].

### 4.1. Zeman UJ90 Melanoma

Gerbils have also played a special part in the history of research carried out at the Department of Biophysics of the Jagiellonian University in Krakow. In the 1990s, in the animal facility of the Department of Biophysics of the Jagiellonian University in Krakow, a melanotic tumor was found on the ear of one animal from a group of Mongolian gerbils, previously treated with N-ethyl-N-nitrosourea. This compound has been described in the literature as a carcinogen with a particular affinity to cells of neural origin [159], showing a preference for places rich in pigment, hairy areas, and those often exposed to the sun (paws, ears, and tail). It can induce tumors in 40% of gerbils exposed to that carcinogen [160]. The tumor was transplanted into other related animals of the breeding stock, which had been maintained by inbred crossing. Thus, a new transplantable Zeman UJ90 melanoma line in gerbils was stabilized [7]. This transplantable melanoma line was used to carry out in vivo Electron Paramagnetic Resonance (EPR) experiments. In contrast to the golden Syrian hamsters, gerbils have a long tail that can be placed inside a resonant cavity, similar to mouse tails inoculated with melanoma. That observation was a good starting point for further extensive research, most of which has been published, and is briefly reported below.

As a desert animal, the Mongolian gerbil comes from a microbiologically pure environment and reveals an impaired immunological reactivity to some immunological stimuli. It is manifested by a weak response of the animal’s macrophages to latex particle challenge [161], and also by weak graft-versus-host [162] and mixed lymphocyte responses from allogenic mixed cultures [163]. Those features may additionally suggest a weak reactivity of the NK cells, and also a low histocompatibility variability, resulting from a high inbreeding level in their breeding stocks. It turned out that the animals revealed a weak response of iNOS (inducible nitric oxide synthase), manifested in EPR studies by showing a low levels of nitric oxide and their hemoglobin complexes (nitroso-hemoglobin, HbNO) in tumors growing in situ. That was entirely different from other animal tumors studied by us, but similar to human tumors [164,165]. As solid tumors of BHM revealed the HbNO signal, its lack of expression in gerbil tumors must have been a result of low iNOS activity rather than the low polymorphism of histocompatibility genes. The EPR signal of HbNO in Zeman UJ90 tumors could be induced only by a strong immunological stimulus—lipopolysaccharide (LPS) [166,167]. The existence of amelanotic sub-line (see further) made it possible to demonstrate the time- and dose dependence of the HbNO EPR signal intensity on LPS, and its inhibition by analogs of L-Arginine, the substrate for NO synthesis by iNOS [168]. That extraordinary property of those laboratory animals could also be demonstrated by a slower and lower response of gerbils to xenotransplants of rat heart tissues, and a weaker dependence on pre-sensitization with donor splenocytes [169]. The ability to induce NO synthesis by LPS served to demonstrate an in vivo production of NO by spin-trapping at S-band, which was followed by in vivo observations of NO production in melanoma in situ. These findings were observed in animal (gerbil and mouse) tails placed inside the resonant cavity [170].

**Figure 7:**
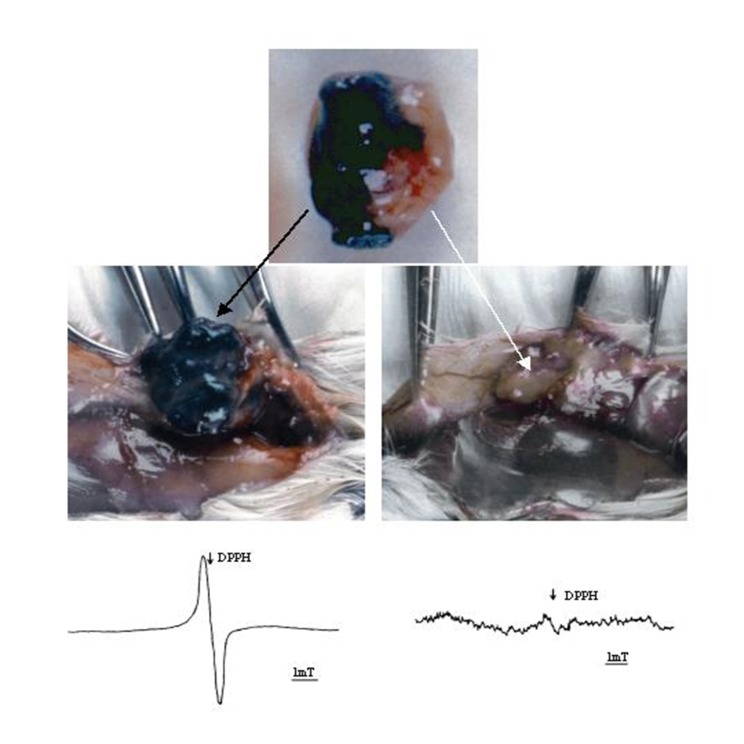
Electron paramagnetic resonance (EPR) analysis of melanotic (**left** EPR spectrum) and amelanotic tumors (**right**) of Zeman UJ90 melanomas, corresponding to the black and white tumors (photographs in the middle row). The white tumor was obtained from the white parts of the two-color tumor which appeared in passage 7 (upper photograph). DPPH: the position of a free radical signal (g = 2.0037). Reprinted with permission from Copyright 2003 John Wiley & Sons Ltd. [8].

Initially, the Zeman UJ90 line was heavily pigmented and grew slowly. After the fourth passage, a rapid acceleration of growth was observed and the FGM (Fast Growing Melanoma) sub-line emerged. During the seventh passage, a part of the growing tumor was completely devoid of pigment. A second amelanotic sub-line (A-FGM) was derived from that fragment (Figure 7). In the case of the Zeman UJ90 melanoma, the non-pigmented sub-line has a greater capacity to create metastases (in 80% of the implanted animals) in comparison to the pigmented sub-line (33% animals) [7]. The observed phenomenon stands in contrast with the case of Bomirski hamster melanoma, where a higher metastatic capacity was reported for the heavily pigmented BHM Ma line [5]. Comparison of the regression line slopes revealed that the rate of growth of the A-FGM sub-line was about three times lower than that of the melanotic M-FGM sub-line, which was consistent with the observed difference in tumor size measured after the experiment. This change towards a slower growth rate of the non-pigmented amelanotic line was also unexpected, as experimental amelanotic tumors tend to grow faster (less differentiated), being better oxygenated and nourished (better blood-supply) than melanotic ones [6,127,171,172]. However, this is not always the case, as shown for the hypomelanotic BMH MI, which grows slower than the more pigmented Ma melanoma [126]. Selection of BHM MI for less and more pigmented variants generated sub-lines with significantly different levels of melanin and tyrosinase, but without any significant effect on growth rate [127].

### 4.2. Irradiation of Zeman UJ90 Melanoma

The melanotic and amelanotic versions of Zeman UJ90 melanoma turned out to be interesting models to determine tumor sensitivity to X radiation, and they were exposed to low radiation doses. The response to the doses of 5 and 10 Gy was weak, which was not surprising. Radiation has an inhibitory effect on tumor growth: both the pigmented and the non-pigmented forms grew slower after irradiation than in the control arm. The effectiveness of X radiation increased linearly with the dose applied, which was expected. Unexpectedly, melanin did play a radioprotective role for the tumor, unlike in the case of hamster tumors. However, that was not a unique or isolated phenomenon [57].

The radiological phenomena associated with Zeman UJ90 melanomas are congruent with earlier observations on a general radioresistance of Mongolian gerbils [173,174,175,176]. All that makes the Mongolian gerbil a really unique laboratory animal in the context of melanoma radiotherapy studies. In this context, one can conclude that the actual radioprotection of melanin strongly depends on the model involved, and in clinical practice on the particular case in question and the stage of tumor development [57,177].

## 5. Human Melanoma

### 5.1. Radiosensitivity of Human Skin Melanoma/Why Is Radiation Not Used in the Treatment of Human Skin Melanoma

Prevention, early diagnosis, and surgical excision of the tumor, when the disease is localized to the skin, represent the golden standards of melanoma management [178,179]. Recent advances in melanoma therapy have led to a successful use of targeted therapy or therapy based on modulations of immune responses, while managing stage III–IV disease [180,181,182]. Although such strategies are associated with adverse effects, financial costs, and development of tumor resistance mechanisms resulting in recurrent disease and ultimate death (discussed in [82,182,183,184,185,186]), systemic strategies are preferred in most clinical settings.

The past reluctance to apply radiotherapy in melanoma treatment was secondary to the opinion that melanomas in general were resistant to radiation [187]. However, significant evidence has been accumulated, indicating that melanomas have a wide range of sensitivity to radiation [187,188]. Currently, radiotherapy is used in selected patients with lentigo maligna melanoma, and as an adjuvant or palliative approach in selected patients with regional or systemic metastatic disease [189,190,191,192,193,194]. It must be noted that some authors recommended caution in the use of adjuvant radiotherapy that should be reserved for high-risk patients, because of its negative impact on overall survival [192]. Interestingly, beneficial effects of adjuvant radiotherapy have been documented for desmoplastic, lentigo maligna, and mucosal melanomas [192,193,194,195,196,197,198,199,200,201]. The use of radiotherapy in the treatment of lentigo maligna may require some selectivity, because of the superiority of the surgical approach [202] and an attractive alternative, i.e., topical treatment with imiquimod [203]. The most promising are the effects of radiotherapy in adjuvant treatment of desmoplastic melanomas [193,195,196,204] that are devoid of melanin pigment [179,202].

Therefore, it can be safely concluded that radiation should be considered as an adjuvant therapy, depending on the context. Also, the presence or lack of melanin pigmentation should be considered, when selecting different therapeutic options, because melanogenesis may affect the behavior and metabolic status of melanoma cells [15,19,92,108,205,206]. The latter consideration is in agreement with recent clinical and pathological studies that have demonstrated that the presence of melanin in metastatic melanomas attenuated the positive outcome of radiotherapy [177].

### 5.2. Radiosensitization of Melanoma Cells through Inhibition of Melanoma Pigmentation

Experimental, cell culture-based studies showed radioresistance of melanoma cells, but the first results related to the sensitivity of human melanoma cell lines with different pigmentation levels to ionizing radiation were contradictory. While Kinnaert et al. [207] found that non-pigmented melanoma cells had a significantly lower resistance to X radiation than the pigmented ones, Barranco et al. [208] observed a high radioresistance of melanoma cells, independent of their pigmentation levels, in three different melanoma cell lines. However, those discrepancies could have arisen from different genotypes of the melanoma cell lines under investigation. Our study, using one line of melanoma cells SKMel-188, with different melanogenesis dependent on the melanin precursor levels in the medium [209], eliminated the impact of genomic differences on the radiosensitivity of melanoma cells [210]. The human melanoma cell line SKMel-188 is characterized by inducible melanogenesis. Under the conditions of low levels of melanin precursors in culture medium melanoma cells remain amelanotic, whereas when cultured in the presence of high levels of melanin precursors, the cells become melanotic [92,108,206,209,211]. Pigmented SKMel-188 melanoma cells showed higher viability after gamma irradiation and increased melanogenic activity was positively correlated with melanoma cell viability after irradiation with 15 Gy gamma radiation (*r* = 0.8, *p* < 0.0001). Tyrosinase activity inhibition with N-phenylthiourea or copper-chelating agent, d-penicillamine, resulted in an increased sensitivity to gamma radiation, and decreased survival after irradiation [210]. Interestingly, the same approach might potentially sensitize melanoma cells to chemo- or immunotherapy [15,82,206,212].

Our recent clinical-based study showed that melanogenesis in human cutaneous primary metastasizing melanomas (stages III and IV), and in lymph node melanoma metastases, was related to a shorter overall and disease-free survival [16]. A subsequent analysis revealed that melanoma patients with amelanotic metastatic tumors showed significantly longer survival after radiotherapy, and longer overall survival time than patients with pigmented tumors, who received either radiotherapy or chemotherapy and radiotherapy [177]. Additionally, Shields and co-authors observed that the presence of melanin was found to be an unfavorable marker of metastasis and death in a multivariable analysis of ciliary body and choroidal melanomas [213,214].

Thus, the experimental and clinical data indicate that inhibition of melanogenesis could be used for the radiosensitization of melanoma cells to ionizing radiation to improve melanoma radiotherapy efficacy.

### 5.3. Radiosensitivity of Uveal Melanoma Tumors

#### 5.3.1. Brachytherapy in Clinical Practice

Human melanomas are less radiosensitive as compared with some other neoplasms due to a slower cell turnover, but most of the uveal melanomas (UM) show satisfactory regression after radiotherapy. Radiotherapy of intraocular melanoma is a therapeutic method used next to surgery. Both external beam and brachytherapy (plaques) are used. Plaque brachytherapy (ruthenium or iodine) is the most common conservative treatment in the management of choroidal melanomas, followed by proton beam radiotherapy. Brachytherapy has been used to treat intraocular tumors since 1930. Consecutive publications report ^60^Co, ^106^Ru, ^125^I, ^103^Pd, ^90^Sr, and ^130^Cs sources [215]. The most commonly used are Ru (beta emitter, recommended for small and medium-sized tumors) and I plaques (gamma irradiation with deeper penetration). The prescription dose range is 70–100 Gy [216]. The COMS (Collaborative Ocular Melanoma Study) study compared enucleation to ^125^I brachytherapy in medium-size tumors. There was no difference in melanoma-associated and overall-cause mortality between the two treatment modalities. The COMS study was restricted to the use of ^125^I plaques [217]. The five-year local control rates after brachytherapy averaged at 89.5% (range 69.9–97.9%). The recurrence rate following ^106^Ru brachytherapy was 3–16% in various studies [217]. However, brachytherapy also affects the intraocular structures, sclera, ocular muscles, conjunctiva, corneal surface integrity, tear production, eyelashes, and eyelids. Within the eye, radiation can cause cataract, retinopathy, optic neuropathy, hemorrhage, retinal detachment, neovascularization, and secondary glaucoma. The side effects involved may result in severe deterioration or the loss of vision. Results of various studies [218] using different analytical techniques and visual acuity endpoints, have indicated that visual acuity is generally preserved in patients with smaller uveal melanoma situated farther from the optic disc and fovea. At 10 years’ follow-up, 68% of patients demonstrated poor visual acuity [218].

#### 5.3.2. Proton Beam Radiotherapy (PBRT) of Uveal Melanoma

Teleradiotherapy is the second method of radiotherapy, next to brachytherapy, dedicated to uveal melanoma patients. PBRT has been the most commonly used option amongst all types of teleradiotherapy, since the 1970s. Charged particle therapy of uveal melanoma is successfully applied in many clinical centers around the world. PBRT is characterized by a very precise dispersion of radiation that enables destruction of the targeted neoplastic tissue at various depths in the body.

Proton beam radiotherapy is particularly dedicated to lesions located close to the optic disc and macula [216,217,218,219,220,221,222]. In intraocular neoplasms, proton beam radiotherapy has the same effect on the survival rate as brachytherapy [216,217,218,219,220,221,222].

Verma et al. summarized fourteen original investigations from 10 different institutions, conducted from 2000 to 2015. In that analysis, five-year local control rates exceeded 90%, which persisted at 10 and 15 years. Five-year overall survival rates ranged from 70% to 85%, five-year metastasis-free survival and disease-specific survival ranged from 75% to 90%, with a more recent series reporting higher values. With the removal of smaller studies, five-year enucleation rates were consistently between 7% and 10%. Many patients (60–70%) showed a post-PBRT visual acuity decrease, but still retained purposeful vision (>20/200) [223].

The above results are comparable with our outcomes. We observed a 93.3% local control rate, and deterioration of visual acuity in 60% of patients. Complications were observed in 31.5% of cases (dry eye syndrome, glaucoma, cataract, retinopathy, maculopathy, and neuropathy). Enucleation was performed in 2.8% of the cases, due to a massive melanoma relapse or neovascular glaucoma with a massive vitreous hemorrhage [221,224].

Proton beam radiotherapy enables a very high local tumor control, and preservation of the eyeball in many cases, with visual acuity depending on the tumor size and location.

#### 5.3.3. Proteomic Study of Human Skin Melanoma Cells (BLM) Treated with Proton Beam Irradiation

Proteomic analysis of the BLM melanoma cell line irradiated with a low dose of 3 Gy of proton beam shows a significant (more than 1.5× change) upregulation of 13 proteins and downregulation of four proteins [225]. These proteins might be roughly grouped into four categories by their function: (i) DNA repair and RNA regulation (VCP, MVP, STRAP, FAB-2, Lamine A/C GAPDH); (ii) cell survival and stress response (STRAP, MCM7, Annexin 7, MVP, Caprin-1, PDCD6, VCP, HSP 70); (iii) cell metabolism (TIM, GAPDH, VCP); and (iv) cytoskeleton and motility (Moesin, Actinin 4, FAB-2, Vimentin, Annexin 7, Lamine A/C, Lamine B). Of particular interest is the substantial decrease (2.3×) in vimentin, a marker of EMT and of the metastatic properties of melanoma [226]. Future works will include other cancer lines, such as uveal melanoma or prostate cancer, both of which respond well to proton beam therapy.

#### 5.3.4. Radio-Phototherapy of Uveal Melanoma

Indocyanine green (ICG) photodynamic therapy administered with brachytherapy was tested in a clinical setting, involving 38 patients [227]. The baseline ICG study showed pathological intrinsic vasculature in all examined cases. Six months after the indocyanine-PDT treatment, changes in microcirculation were detected in all cases as well as a significant decrease in tumor thickness in ultrasonography (mean 38%). A complete regression of intrinsic vessels was demonstrated by indocyanine green angiography in 26 cases, and partial regression of pathological vascularization was found in 12 patients. In the Campagnoli study, involving five patients with amelanotic choroidal melanoma treated with PDT, four patients did not respond to treatment [228]. They concluded that radiotherapy was the main damaging agent, with phototherapy effects considered negligible. Contrary to the above, verteporfin-PDT as primary treatment in small choroidal melanomas resulted in an 80% rate of local tumor control [229], which slightly lower than in classical brachytherapy.

PDT treatment of skin melanoma was reviewed and its efficacy was dependent on the photosensitizer used, and moderate effects were seen when PDT was used in combination with immunotherapy, but not with radiation [230].

#### 5.3.5. Radio-Chelation Therapy in Clinical Trials

Cuprenil and Chelaton (Polfa) were used as radiosensitizers in patients treated with ^60^Co gamma rays (brachytherapy) for choroidal melanoma in a pilot study. Chelators were administered for 7–10 days before, and 9–14 days during the brachytherapy. Tumor size was determined 8 weeks and 16 weeks after treatment. Tumor size volume decreased approximately 45% at 16 weeks after Cuprenil treatment. The sensitizing action of chelators may be due to the inhibition of oxygen consumption in melanotic cells by those compounds [231], and inhibition of melanogenesis as shown in other models [206,210].

## 6. Conclusions

Since malignant melanomas are responsible for the highest mortality rate among patients with skin cancers, and exhibit a high incidence rate in the white population, it is of utmost importance to develop and test multiple therapeutic strategies, using appropriate animal models. This is crucial for advanced melanomas at the vertical growth phase or metastatic disease. An impressive progress has been made in the development of new strategies in targeted therapy and immunotherapy and other treatment modalities. However, there is a lack of optimism, with respect to long-term survival of melanoma patients, because of the pre-existing or acquired resistance developing to the applied therapies.

Radiotherapy is used in the intraocular melanomas with satisfactory outcomes. Both external beam and brachytherapy are utilized in the treatment of uveal melanomas. For brachytherapy, ^60^Co, ^106^Ru, ^125^I, ^103^Pd, ^90^Sr, and ^130^Cs are the available radiation sources, with the most commonly used beta emitter being ^106^Ru, and gamma irradiation (^125^I) preferred for tumors with deeper penetration. Proton beam radiotherapy constitutes the second method of radiotherapy that enables efficient local tumor control with relative preservation of the eyeball, depending on disease progression. Radiotherapy is rarely used in cutaneous melanomas, except for a palliative approach in selected patients, likely due to the long-held opinion that melanomas are resistant to radiation. The resistance may in part be explained by the radioprotective properties of melanin. Interestingly, desmoplastic melanomas, which are amelanotic, are responsive to radiotherapy. Therefore, radiotherapy represents a viable alternative in the treatment of melanomas, depending on the tumor phenotype and location.

The discussed models of rodent melanoma, namely the Bomirski and Zeman melanoma lines transplantable in hamsters and gerbils, respectively, constitute remarkable animal models to study and test different radiotherapeutic approaches before clinical treatment of stage 3 and 4 disease. Specifically, non-pigmented BHM melanoma is responsive to radiotherapy, either after subcutaneous transplantation or implantation into the eye. Similarly, Zeman melanoma is radiosensitive, but independently on pigmentation, while pigmented BHM melanomas are markedly more resistant to X irradiation than non-pigmented ones. Moreover, high-LET radiation (fast neutrons) is effective in the inhibition of BHM tumor growth independently on its pigmentation.

Thus, Bomirski hamster and Zeman gerbil melanomas represent a comprehensive set of preclinical models—resistant and sensitive to therapies, allowing to define optimal conditions for radiotherapy such as inhibition of melanogenesis in *X*-ray therapy or selection of proper radiation, which would efficiently treat tumors independently of the level of pigmentation.

## Figures and Tables

**Table 1:** Pathobiological parameters transplantable melanotic (Ma) and amelanotic (Ab) lines of Bomirski hamster melanoma model.

	Melanotic Melanoma Ma	Amelanotic Melanoma Ab	Reference
Origin	Developed as a spontaneous malignant melanoma of the skin.	Developed as a spontaneous alteration of Ma melanotic melanoma.	[4,5]
Year of origin	1959	1963	[4]
Amount of tissue needed for 100% transplantability (mg)	200	50	[4,5]
Transplantation interval (days)	21 ± 3	12 ± 2	
Survival time of implanted hamster (days)	81 ± 5.8	27 ± 1.5	[5]
Most frequent locations of metastases	Lungs, lymph nodes	Kidneys, liver, lymph nodes	[4]
Histological and ultrastructural features			[4,110]
	Epitheloidal cells	Polygonal cells	
	Melanosomes and premelanosomes	Lack of melanin and melanosomes	
	Golgi area is moderately developed	Golgi area is more extensive than in Ma; Products of tyrosinase activity accumulate in the vesicles of the trans-GA	
	Moderate amount of ribosomes	Abundant ribosomes	
	Mitosis is rare	Mitosis is frequent	
	RER and SER are moderately developed	RER and SER are very well developed	
	Some mitochondria	Some mitochondria	
Plasma membrane structure:			
1. Carbohydrates content (nmol/mg of protein)	1702	631	[111]
2. Heterogeneity	3 protein fractions 6 glycoprotein fractions	1 protein fraction 8 glycoprotein fractions	[112]
3. Membrane fluidity and molecular mobility in the plasmatic membrane		Lower degree of order in the phospholipid bilayer; increase in membrane fluidity	[113]
4. Expression of P glycoprotein (Pgp)	70% of cells Pgp positive	10% of cells Pgp positive	[114]
5. Ganglioside content	High level of GM3 Low level of GD3 and 9-*O*-acetyl-GD3	Low level of GM3 High level of GD3 and 9-*O*-acetyl-GD3	[115,116]
6. Neutral glycolipid content	High level of GL1	High level of Gb3, Gb4, Gb5	[117]
Antigenicity	Low	Increased in comparison to Ma	[118]
Immunogenicity	Low	Increased in comparison to Ma	[119,120]
Cytokine secretion		Altered secretion of IL-6, IL-10, TNF-α	[121]
DNA ploidy	4n	3n	[5,122]
Radiosensitivity	Low	High	[105]
Ability for apoptosis	High propensity for spontaneous apoptosis	Low endogenous apoptosis but highly sensitive to camptothecin-induced apoptosis	[103,104]
Cell cycle analysis	30% in S + G2/M phase	40% in S + G2/M phase	[104]
Main biochemical features			
1. Tyrosinase activity	High tyrosinase activity	Low tyrosinase activity	[123,124]
2. Glycolysis		High aerobic and anaerobic glycolysis	[84]
3. Antioxidant enzymes	High activity of dismutase/peroxidase	Relatively low activity of dismutases/peroxidase	[125]
4. Mitochondrial transmembrane potential ΔΨ	Relatively low	Relatively high	[109]
5. Oxygen consumption	Relatively high	Relatively low	[106]
6. Enzyme activities	Relatively high activities of citrate synthase, succinate dehydrogenase, malate dehydrogenase higher than in Ab	Relatively high activities of NAD-dependent glycerol-3-phosphate dehydrogenase higher than Ma	[106]
